# Misleading subcutaneous mycosis: a case report of subsequent clinical mycetoma-like and histological chromoblastomycosis-like lesions

**DOI:** 10.1590/S1678-9946202466034

**Published:** 2024-06-07

**Authors:** João Paulo Turri Brufatto, Laís Pontes, Angélica Zaninelli Schreiber, Maria Leticia Cintra, Cintia Avila Souza, Luciana Vilela Gomide, Helena Maciel Mendonça Tolentino Guerra, Rafael Fantelli Stelini, Isabela Vilela Brum, Andrea Fernandes Eloy da Costa França, Renata Ferreira Magalhães, Paulo Eduardo Neves Ferreira Velho

**Affiliations:** 1 Universidade Estadual de Campinas, Faculdade de Ciências Médicas, Departamento de Dermatologia, Campinas, São Paulo, Brazil; 2 Universidade Estadual de Campinas, Faculdade de Ciências Médicas, Departamento de Patologia, Campinhas, São Paulo, Brazil

**Keywords:** Diagnosis, Chromoblastomycosis, Hyalohyphomycosis, Phaeohyphomycosis, Mycetoma

## Abstract

Hyalohyphomycosis and phaeohyphomycosis are groups of mycoses caused by several agents and show different clinical manifestations. We report a case of an immunocompromised patient who presented rare manifestations of opportunistic mycoses: mycetoma-like hyalohyphomycosis on his right foot caused by *Colletotrichum gloeosporioides*, followed by cutaneous phaeohyphomycosis on his right forearm caused by *Exophiala oligosperma*. Further to the rarity of this case, the patient's lesion on the foot shows that the clinical aspects of mycetomas could falsely appear in other fungal infections similar to hyalohyphomycosis. We also show that the muriform cells that were seen in the direct and anatomopathological examination of the skin are not pathognomonic of chromoblastomycosis, as observed in the lesion of the patient's forearm.

## INTRODUCTION

We report the case of a 61-year-old white immunocompromised man with subsequent hyalohyphomycosis and phaeohyphomycosis, both with positive responses to treatments. Hyalohyphomycosis and phaeohyphomycosis are groups of mycoses caused by several agents and show different clinical manifestations^1-3^. The genus *Colletotrichum*, detected in the patient's foot, usually infect plants, rarely affecting humans^2^. It is frequently related to inoculating organic or woody material into the eye or cutaneous surfaces, causing hyalohyphomycosis^2^. The genus *Exophiala*, isolated from his forearm, is one of the causes of phaeohyphomycosis^4^. A common manifestation of dematiaceous fungi is a superficial localized cutaneous or subcutaneous disease. Solid organ transplant recipients have increasingly reported infections caused by these organisms. In certain studies, up to 65% of dematiaceous fungal infections have been reported in organ transplant recipients^3^. This case demonstrates two infections that were not what they appeared to be: a lesion on the left foot that clinically resembles a mycetoma but without grains, as attested via physical examination or histology, and a lesion on the right forearm with incompatible clinical aspects with chromoblastomycosis, despite the anatomopathological histology finding muriform cells. The clinical presentation, causative species, treatment, and the patient's lesions progression support the diagnosis of hyalohyphomycosis on the left foot and phaeohyphomycosis on the right forearm.

### Consent for publication

The reported work was conducted following the World Medical Association's Code of Ethics (Declaration of Helsinki). The patient signed an informed consent form.

## CASE REPORT

**Figure 1:**
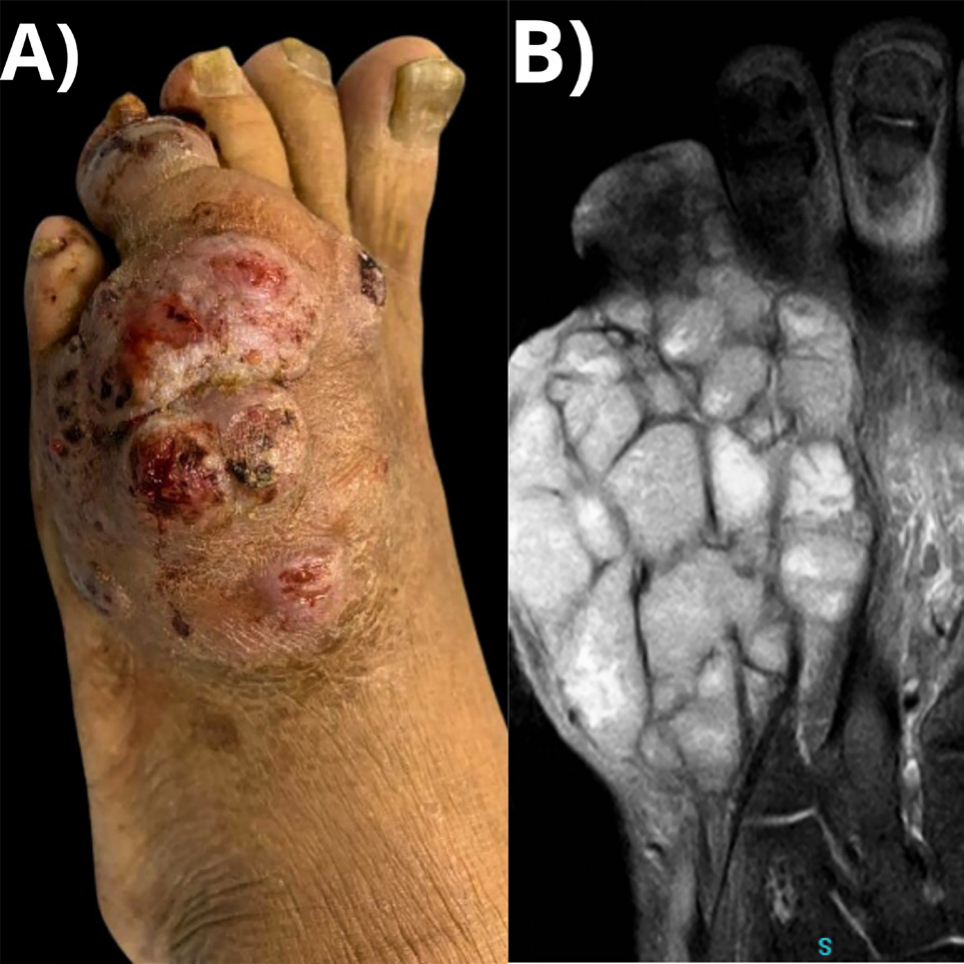
A) Tumor with lobulated surface and fistulous ostia, with drainage of serosanguineous material, without grains, located on the dorsum of the left foot; B) Magnetic resonance imaging depicting multiple septa and nodules affecting deep tissue planes, containing exudate, without bone involvement.

**Figure 2:**
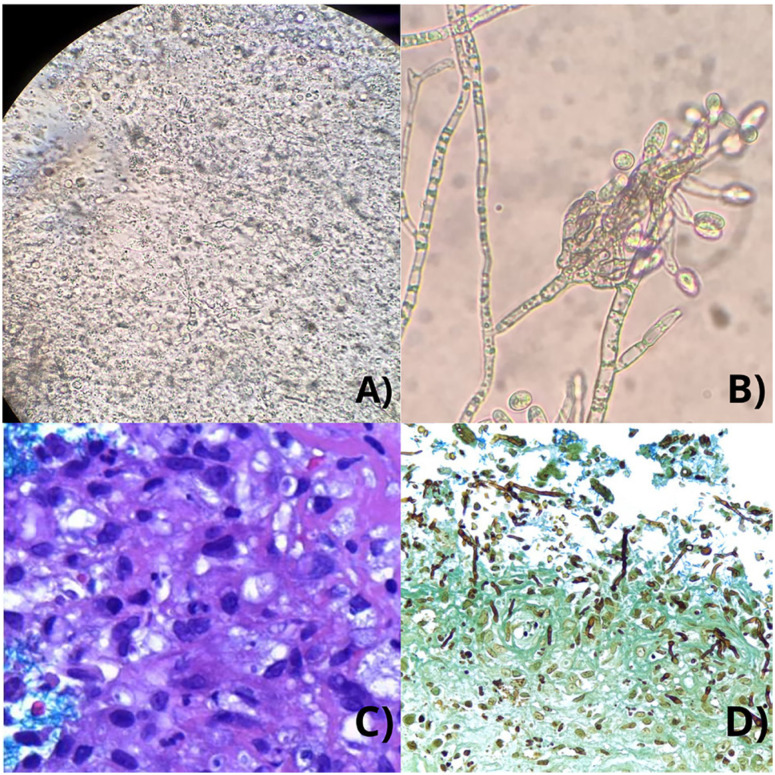
A) Direct examination of skin scrapings in Potassium Hydroxide (KOH) 20%, showing filamentous hyphae; B) Characteristic structures of the *Colletotrichum gloeosporioides* complex at 40× magnification.; C) Chronic granulomatous suppurative dermatitis presenting frequent filamentous fungal structures, without grains, observed in Hematoxylin-eosin staining at 400× magnification; D) Grocott's methenamine silver staining of sinus mucosal biopsy demonstrating diverse fungal elements, including broad, pauci-septate hyphae (5–10 µm), suggestive of mucormycosis, and thinner hyphae, mostly with right-angle branching, indicative of hyalohyphomycosis

A 61-year-old farm worker Brazilian man was admitted to the dermatological outpatient clinic in 2019 with progressive growth of a cutaneous lesion on the left foot after local trauma caused by a plant during the past year. On examination, a skin lobulated tumor was noted on the dorsum of the left foot, with the presence of fistulous ostia and drainage of serosanguineous material (Figure 1A). A magnetic resonance imaging (MRI) examination of the affected foot was performed for better therapeutic planning (Figure 1B). His past medical history was remarkable for a previous kidney transplantation in 2014, for which he was taking prednisone 10 mg and azathioprine 100 mg daily. Tests for human immunodeficiency virus (HIV), syphilis, hepatitis B, and C were nonreactive throughout the follow-up. A chronic granulomatous suppurative dermatitis with frequent filamentous fungal structures was observed on the histological sections (Figures 2C and 2D). Direct analysis with potassium hydroxide revealed septate hyaline hyphae. Samples were cultured on potato dextrose agar (PDA) and subjected to mass spectrometry technique, finding positive results for *Colletotrichum gloeosporioides* species complex (Figures 2A and 2B) ^2^. The patient was initially treated with voriconazole 400 mg/d for 14 days, followed by itraconazole 200 mg/d as monotherapy with a complete resolution of the lesion after three months.

**Figure 3:**
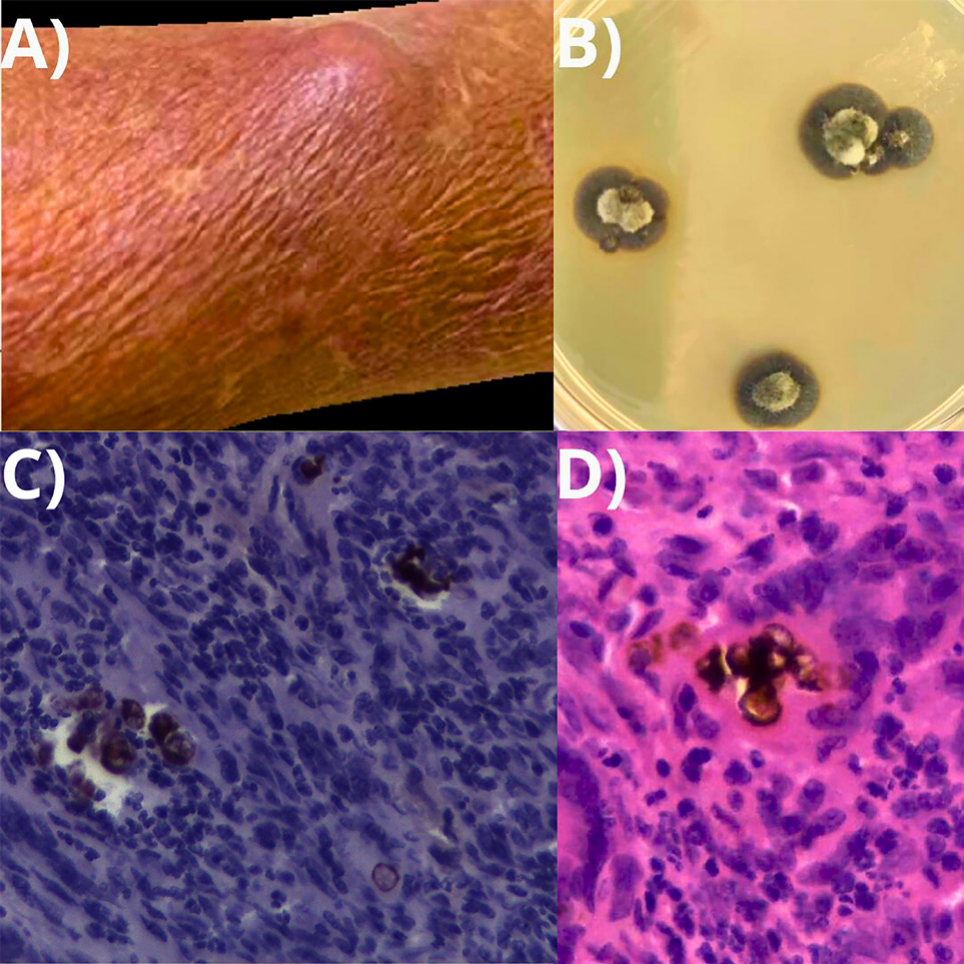
A) Nodular lesion measuring 1.5 cm in diameter, located on the right upper forearm; B) Colonies of *Exophiala oligosperma* complex cultured on Saboraud Dextrose Agar after 2 months of growth; C) Muriform cells observed in Periodic acid–Schiff (PAS) staining at 400× magnification; D) Muriform cells observed in hematoxylin-eosin staining at 400× magnification.

Despite using itraconazole 200 mg/day, the patient developed, in 2021, a nodular lesion, nonadherent, on the right upper forearm, which evolved after one year, growing to 1.5 cm in diameter (Figure 3A). Interestingly, the patient reported a scar close to the new lesion from a previously performed surgical procedure. At that time, he was taking tacrolimus 5 mg/day and prednisone 5 mg/day for kidney transplantation. Direct microscopy research of this new lesion showed dematiaceous fungi isolated from microculture (Figure 3B) and fungal structures suggestive of muriform cells in a skin fragment histology (Figures 3C and 3D). Surgical excision of the lesion was performed, with an AP examination showing chronic granulomatous dermatitis with microabscesses, muriform cells, and exuberant epidemic hyperplasia. In the culture, it was possible to observe a microorganism with black colors, which was subjected to the sequencing of the ITS4/5 genes, showing the agent *Exophiala oligosperma* ^5^.

The patient was investigated with whole-body computed tomography for distant mycological involvement, but no distant infection was detected. The itraconazole dose was doubled to 400 mg per day, with no lesion recurrence after five months of treatment. The patient continued using itraconazole 200 mg daily for seven months after that, and treatment was discontinued in May 2023. After a 12-month follow-up, the patient presented no relapses. Therefore, the patient was diagnosed with phaeohyphomycosis due to the absence of clinical aspects of the lesion compatible with chromoblastomycosis and the positive response to itraconazole.

## DISCUSSION

We report a case of an immunocompromised patient who had rare manifestations of opportunistic mycoses: hyalohyphomycosis on the right foot caused by *Colletotrichum gloeosporioides* followed later by cutaneous phaeohyphomycosis on the right forearm caused by *Exophiala oligosperma* during itraconazole 200 mg daily therapy.

Fungi can emerge in many forms, such as endosporulation spherules, hyphae, budding yeast, or a combination of these forms^6^. The tissue cells of hosts react according to immune system, virulence, duration, and type of fungi^6^. Isolation is an important method for an accurate diagnosis of subcutaneous fungal infections^7^. It is a major method for diagnosing fungal infections, similar to specific antibody detection, antigen detection, or PCR technique^8^. Subcutaneous mycoses are a type of fungal infection that more prevalently affects immunosuppressed patients and travelers, and it is gaining importance due to the increase in cases over the years^1^.

The term hyalohyphomycosis accommodates mycotic infections, in which the etiological agents’ tissue shows septate hyphae without pigment on the cell wall^9-11^. In another view, mycetoma is a chronic suppurative skin infection characterized by a symptomatic triad: swelling of the affected area, multiple sinus formation, and a purulent discharge containing clinical or histological grains^12-14^. The patient in the case presented no clinical or histological grains. In addition, we had the isolation of *Colletotrichum* sp. in the culture. Currently, the *Colletotrichum gloeosporioides* species has been described as a hyaline fungus with typical features of hyaline hyphae under proper examination^2^. We observed this same pattern of hyphae, as can be seen in Figures 2A and 2B. Therefore, our team recognized that it was a hyalohyphomycosis on our patient's foot.

On the other hand, phaeohyphomycosis and chromoblastomycosis are characterized by the presence of dematiaceous fungi under proper examination, which are pigmented filamentous fungi that contain melanin in their cell walls^15,16^. Chromoblastomycosis typically shows muriform cells in histology^3^. This structure serves as a residence mechanism, reducing the effectiveness of medications such as azoles^3^, and is usually recognized as a pathognomonic finding of chromoblastomycosis^3,17^. However, our patient had no typical chromoblastomycosis lesions, such as verrucous and plaque lesions^18^, and presented no common quick clinical response for an itraconazole dose of 400 mg. Zeng and colleagues have already demonstrated the wide clinical spectrum that the genus *Exophiala* can generate and how its species cause types of phaeohyphomycosis with pigmented filamentous hyphae under direct exam^4,19,20^. We observed the same pattern of pigmented filamentous hyphae, as seen in Figure 3B. Based on that, our team recognized that it was a phaeohyphomycosis on the forearm of our patient.

Further to the rarity of the case, the patient's lesion on the foot shows that the clinical aspects of mycetomas could falsely appear in other fungal infections similar to hyalohyphomycosis. We also show that the muriform cells seen in the direct and anatomopathological examination of the skin are not pathognomonic of chromoblastomycosis, as observed in the lesion of the patient's forearm.

## CONCLUSION

We report a case of an immunocompromised patient who presented with a mycetoma-like hyalohyphomycosis on his right foot caused by *Colletotrichum gloeosporioides*, followed later by cutaneous phaeohyphomycosis on his right forearm caused by *Exophiala oligosperma*. Further to the rarity of the case, the first patient's lesion suggests that the clinical aspects of mycetomas could falsely appear in other fungal infections similar to hyalohyphomycosis. We also show that the muriform cells seen in the direct and the anatomopathological examination of the skin are not pathognomonic of chromoblastomycosis, as observed in the patient's second lesion. The clinical presentation, causative species, treatment method, and patient's lesions progression revealed the subsequent diagnoses of hyalohyphomycosis on the foot and phaeohyphomycosis on the forearm.
